# Hemodynamic Evaluation of a Centrifugal Left Atrial Decompression Pump for Heart Failure with Preserved Ejection Fraction

**DOI:** 10.3390/bioengineering10030366

**Published:** 2023-03-17

**Authors:** Navideh Abbasnezhad, Mathieu Specklin, Farid Bakir, Pascal Leprince, Pichoy Danial

**Affiliations:** 1 Arts et Métiers Institute of Technology, CNAM, LIFSE, HESAM University, F-75013 Paris, France; 2 Department of Cardiovascular and Thoracic Surgery, Institute of Cardiology, Pitié Salpêtrière Hospital, Assistance Publique Hôpitaux de Paris (AP-HP), Sorbonne University, F-75013 Paris, France

**Keywords:** heart failure with preserved ejection fraction, centrifugal blood pumps, mechanical circulatory support, computational fluid dynamics, hemocompatibility

## Abstract

This article discusses a new continuous flow mini pump that has been developed to improve symptoms and prognosis in patients with Heart Failure with Preserved Ejection Fraction (HFpEF), for which there are currently no established treatments. The pump is designed to discharge a reduced percentage of blood volume from the left atrium to the subclavian artery, clamped at the bifurcation with the aortic arch. The overall specifications, design parameters, and hemodynamics of this new device are discussed, along with data from in vitro circulation loop tests and numerical simulations. The article also compares the results for two configurations of the pump with respect to key indicators of hemocompatibility used in blood pump development.

## 1. Introduction

HFpEF, or heart failure with preserved ejection fraction, is a type of heart failure in which the heart muscle is stiff and does not relax properly during diastole. As a result, the heart’s ability to fill with blood is impaired, leading to symptoms such as shortness of breath, fatigue, and swelling in the legs and abdomen. Despite the diastolic dysfunction in HFpEF, the ejection fraction (EF) is preserved. This is because the stiffness of the heart muscle helps to maintain a higher end-diastolic volume. In other words, the heart must work harder to fill with blood during diastole, but the increased volume of blood in the ventricles leads to a stronger contraction during systole. This compensatory mechanism helps to maintain the EF despite the diastolic dysfunction. Additionally, patients with HFpEF often have increased left ventricular filling pressures, which can also contribute to the preservation of EF by improving the force of the heart’s contraction. However, this increased pressure can also cause fluid buildup in the lungs, leading to symptoms of heart failure.

Heart failure is a disease that is becoming more frequent as the population ages [1]. Clinically, heart failure with reduced left ventricular ejection fraction or preserved ejection fraction (HFpEF) is distinguished [2,3]. The incidence of HFpEF is continuously increasing and represents up to 50% of heart failure cases (approximately 500,000 people in France). However, unlike heart failure with reduced ejection fraction, the pharmacological treatments for HFpEF, such as conversion enzyme inhibitors, angiotensin II receptor antagonists, β-blockers, and spironolactone, have proven disappointing and have not been shown to improve survival rates [4,5,6,7]. As a result, the mortality rate of HFpEF is high, with a 29% mortality rate in one year and a 65% mortality rate in five years [8]. Therefore, there is a need to develop interventional therapies to treat this frequent and serious condition.

One of the most experienced mechanical solutions for treating heart failure is left heart unloading. The first proposed solution was the creation of a shunt between the left and right atria to unload the left heart. However, in human studies, the placement of an atrial shunt device did not reduce the overall rate of heart failure events or improve health status in the patient population with a heart failure ejection fraction of greater than or equal to 40% [9].

The second structural alternative proposed to improve cardiac compliance and attenuate the increase in left ventricular end-diastolic pressure was pericardectomy [10,11,12,13,14,15]. Borlaug et al. proposed a new technique for percutaneous pericardectomy via a subxyphoidal approach [16]. In this study, the opening of the anterior pericardium decreased left ventricular end-diastolic pressure during exercise in pigs with HFpEF. However, the attenuation of pressures during exercise was modest, and the clinical impact is, therefore, uncertain.

The third option is to install a discharge system, such as a magnetic levitation centrifugal pump, which is about the size of a battery or pacemaker [17,18]. This device can discharge a reduced volume of blood, ranging from 0.05 to 0.50 L/min. However, the effectiveness of this type of device for treating HFpEF has not been extensively studied. Design and analysis studies are needed to evaluate its clinical, biological, and hemodynamic effects and to ensure the reliability of this medical device [19,20,21,22]. If a centrifugal pump is chosen, it will operate at off-design conditions, far from its nominal point, due to the low flow rate required.

In this study, we present the preliminary results of the development of a mini continuous flow pump designed to meet clinical decompression specifications for the treatment of HFpEF. Two pump prototypes were designed and fabricated, differing only in the distribution of blade thickness along the blade span. We present data from in vitro circulation loop tests as well as numerical simulation results and discuss the in silico results of scalar shear stress (SSS), wall shear stress (WSS), and vorticity.

## 2. Materials and Methods

### 2.1. Design Features and Computational Modeling

#### 2.1.1. Geometry and Design Features

**Figure 1:**
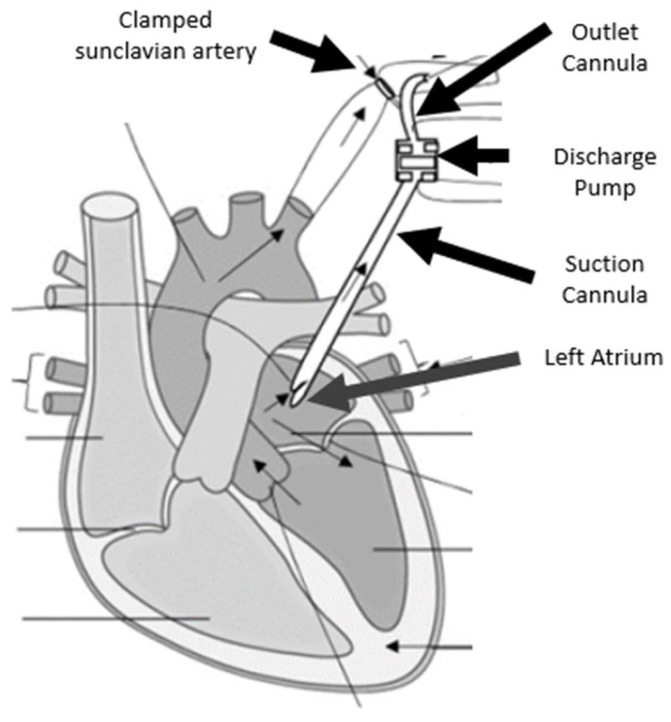
An illustration of how the developed pump will be implanted.

As part of the project, a miniature pump is being developed to continuously eject a small volume of blood from the left atrium to the subclavian artery, which is pinched at the aortic bifurcation (Figure 1).

The specifications for this pump include a centrifugal pump with a volute and a semi-open impeller. The impeller is driven by an electric motor with axial flow and magnetic levitation. The rotational speed should be between 2000 and 5000 revolutions per minute, with a continuous flow rate ranging from 0.05 to 0.5 L/min depending on the required ejection fraction. The pressure gradient should be approximately 80 mm Hg to overcome the pressure loss in the organs supplied by the subclavian artery. It is important to note that the two pump prototypes are not commercially available and will be designed and manufactured in-house.

In this study, two geometric variants of the pump were designed to meet the specifications and they differed in blade thickness. The impellers and volutes were designed using internal 3Dturbo software, V2.0 a comprehensive solution for the design of single-stage centrifugal pumps. This software also allowed for the reverse engineering of centrifugal pumps and facilitated the calculation of performance in design and off-design conditions using a specific 0D/1D solver, as explained in more detail in [23]. The impellers and volutes were then finalized in CATIA.

Apart from a few key dimensions chosen rationally, there are many degrees of freedom available to the centrifugal pump designer to define the other design parameters of the impeller and volute. Therefore, it is necessary to initially choose some design dimensions arbitrarily or intuitively and later verify, through simulations and/or experimental tests, if they meet the requirements.

**Figure 2:**
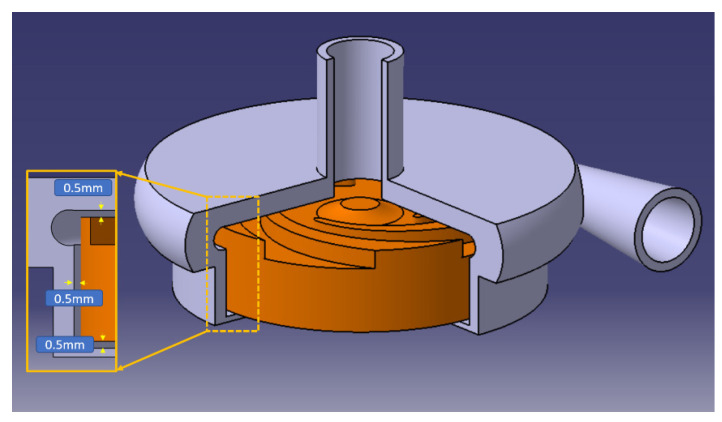
General schema of the pump with the dimensions.

Two impellers, each with 5 blades, were designed with different blade thicknesses, in values and along the span. These impellers have a constant width of 9 mm, with only 2 mm allocated to the blade width and the remaining 7 mm to receive the magnets necessary for electric motorization and magnetic levitation. The main geometric characteristics of the impellers are given in Table 1, and the associated clearance gaps are shown in Figure 2.

**Figure 3:**
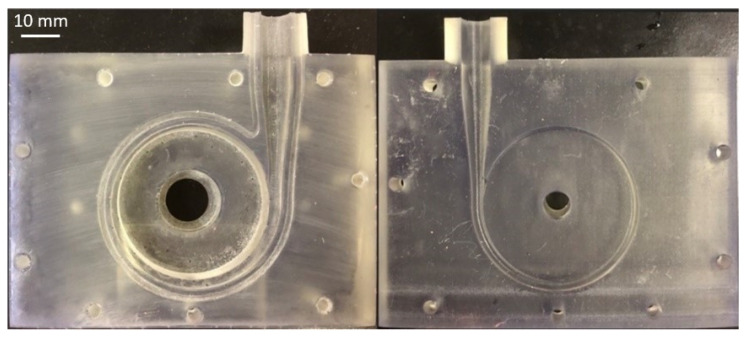
The volute printed by SLA.

Stereolithography (SLA), an additive manufacturing technique, was used to fabricate the components of the two prototypes: a clear resin (product code: FLGPCL04) for the volute and a black resin (product code: FLGPBK04) for the impellers. Figure 3 shows the printed volutes. The resolution in the z-direction used for additive manufacturing SLA was 25 microns.

#### 2.1.2. Fluid Domain and Meshing

**Figure 4:**
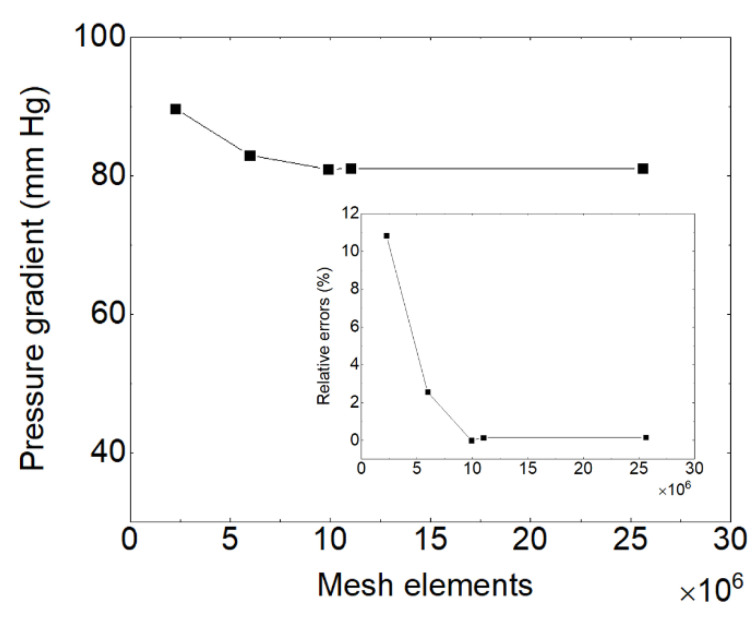
Mesh independence study of different mesh sizes on pressure gradient for the pump with impeller SH1 at the flow rate of 0.5 L/min and rotational speed of 2900 rpm.

**Figure 5:**
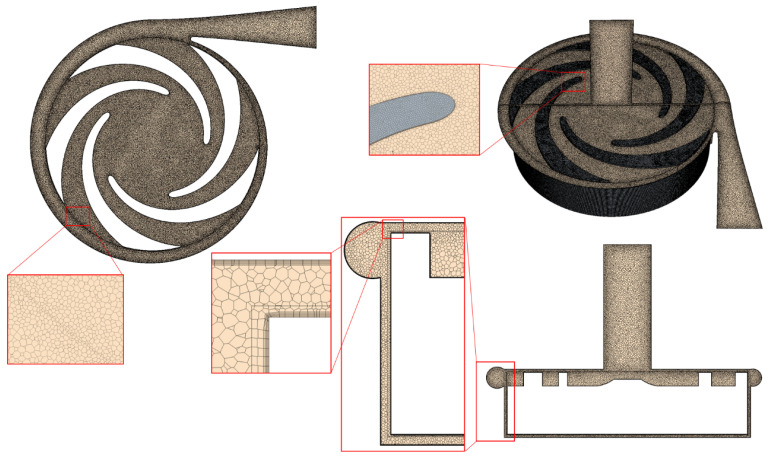
Mesh distribution on the fluid volume of the pump with the impeller of SH1.

The three-dimensional fluid volume was designed based on the geometrical characteristics of both prototypes. Computation was performed using Simcenter StarCCM+ 16.02.009, a software developed by Siemens that employs a Finite Volume approach to discretize the partial differential equations governing fluid behavior. The software uses the SIMPLE (Semi-Implicit Method for Pressure-Linked Equations) algorithm to solve the pressure–velocity coupling inherent in these equations. StarCCM+ can simulate flow in both turbulent and laminar regimes for transient or steady states. Polyhedral non-structured grids were used throughout the computational domains, with finer grids selected in specific areas to obtain more realistic simulation results. Figure 4 shows that, in addition to the polyhedral elements in the core of the domain, six prism layers were added to the walls. In this context, the cell size within the first layer is approximately 0.07 mm, which is significantly smaller than the different gap sizes. Furthermore, it results in an average and maximum y+ of 0.34 and 0.72, respectively, ensuring accurate estimation of velocity gradients at the walls. Different mesh sizes ranging from 1.5 to 13.5 million were created to achieve mesh independence. Figure 4 demonstrates that, when the number of mesh elements exceeds 6 million, the accuracy varies within a 1% range for the pressure gradient, which is monitored as the convergence control variable among other variables. As a result, a final mesh with approximately 10 million elements was adopted for the subsequent computations. Figure 5 displays the mesh of these sections. To perform the 3D CFD computations in a steady state, the Multiple Reference Frames (MRF) method was utilized.

#### 2.1.3. Simulation of the Hemodynamic Characteristics

The numerical simulation models the turbulent, viscous, and incompressible flow field in a 3D domain using the continuity and momentum equations with the k-w SST turbulence model. We have chosen the turbulent flow hypothesis because the Reynolds number based on the blade chord and relative velocity is approximately equal to 5 × 10⁴ at 3000 RPM. Blood is treated as a Newtonian fluid due to the high SSS experienced within the pump [24]. Non-Newtonian behavior can be neglected at SSS rates above 100 s^−1^, where the shear-thinning viscosity reaches a plateau, as is typically the case for existing VADs [24,25,26,27]. Its density and dynamic viscosity are chosen as 1060 kg/m^3^ and 0.0035 Pa.s, respectively. The simulations use a steady-state approach with the impeller domain rotating and all other domains stationary. All physical surfaces are set as no-slip walls, and convergence is achieved when the flow-related residual is below 1 × 10^−6^, and the other residuals are below 2 × 10^−4^.

Regarding the boundary conditions, various references dealing with blood flows in arterial networks, such as [28,29,30,31,32,33,34], have shown that the velocity fields, flow distribution between simulated branches, and magnitude of indicators based on WSS are substantially influenced by the boundary condition strategy adopted. In this work, the pump draws a continuous and incompressible flow of blood from the left atrium under quasi-constant pressure and forces it back into a section of the subclavian artery clamped at the bifurcation with the aortic arch. Therefore, a reference stagnation pressure (*p* = 760 mm Hg) and mass flow rate are, respectively, imposed for the inlet and outlet boundary conditions.

The simulations analyze the hydraulic performance and hemodynamic characteristics of two pumps under different conditions, with impeller rotational speeds ranging from 2000 to 4000 rpm and flow rates ranging from 0 to 2 L/min, with a turbulence intensity of 0.01%.

#### 2.1.4. Hemodynamics and SSS Calculation

One of the major complications of medical circulatory support devices is their tendency to cause hemolysis. This phenomenon is the destruction of the membrane of red blood cells, which results in the release of hemoglobin into the blood plasma. Hemolysis is mainly caused by the duration during which a red blood cell experiences high SSS [24]. One way to limit the onset of hemolysis is to examine the SSS in the flow field, which represents the magnitude of the shear experienced by the blood cells.

Flow fields obtained from computational fluid dynamics (CFD) are used to estimate these hemodynamic quantities and assess hemolysis risks. Shear stress is a second-order tensor whose components are governed by velocity gradients, as follows in Equation (1):

(1) $$\tau_{ij}=-\mu \times \left(\frac{du_{i}}{dx_{j}}+\frac{du_{j}}{dx_{i}}\right)$$

where *μ* is the effective viscosity of the fluid, and the sum of the components represents the effective viscosity.

The SSS then defines the magnitude of these stresses, which is experienced by blood cells, and is defined as [35], Equation (2):

(2) $$\tau =\left[\frac{1}{3}\left\{\tau_{xx}^{2}+\tau_{yy}^{2}+\tau_{zz}^{2}-\tau_{xx}\tau_{yy}-\tau_{yy}\tau_{zz}-\tau_{zz}\tau_{xx}+3\left(\tau_{xy}^{2}+\tau_{yx}^{2}+\tau_{zx}^{2}\right)\right\}\right]{}^{0.5}$$

It is important to keep in mind that the typical SSS observed in the entire circulatory system ranges from 0.1 to 15 Pa [36], and up to 5 Pa for WSS in particular [24]. In this context, medical circulatory support devices lead to SSS levels several times higher than those normally experienced by blood cells. Typical higher thresholds of WSS, corresponding to different blood traumas as described in [37], are approximately 9 Pa, 50 Pa, and 150 Pa. They represent, respectively, von Willebrand factor cleavage and the threshold for platelet activation, both of which increase thrombosis risk, and finally the threshold that usually triggers significant hemolysis. Regarding the latter, ref. [38] found that the hemolysis generated at the outflow grafts of the devices is negligible compared to what is generated inside the device, meaning that the effort must be focused on pump design.

### 2.2. Closed-Loop Test Bench for Hydraulic Performances

**Figure 6:**
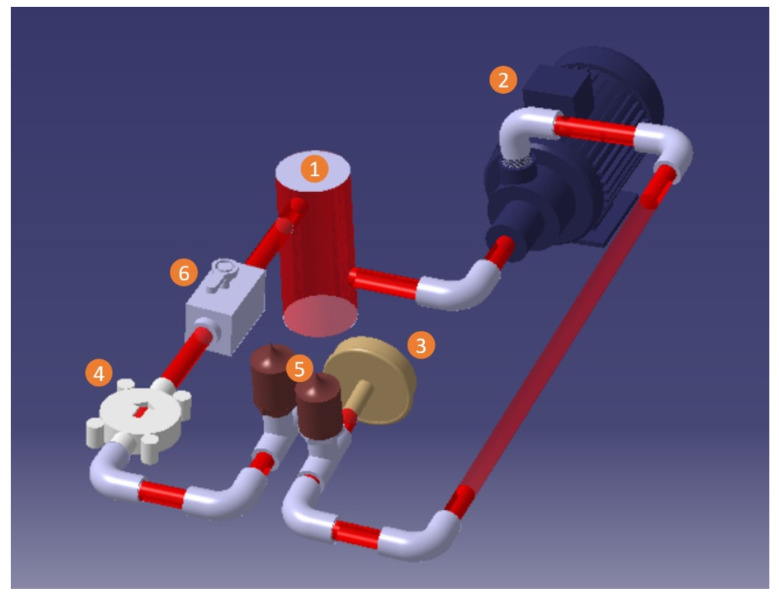
Closed-loop test bench for hydraulic performances: (1) reservoir; (2) circulation pump; (3) prototype-mini pump; (4) flow meters; (5) pressure sensors; (6) valves.

To assess the hydraulic performance of the pump and verify the accuracy of the simulation, a closed-loop test bench was set up. Figure 6 illustrates the configuration of this test bench. The fluid circulating in the system was stored in a one-liter cylindrical plastic reservoir (1). The fluid used was a mixture of glycerol and water, with a ratio of 40/60 at the temperature of 22°C to ensure the same viscosity and density as blood [39,40,41]. A VM-D20T-SP centrifugal pump was connected to the reservoir’s outlet (2) to facilitate circulation. The impeller of the prototype pumps being studied (3) was driven by a Maxon EC-4-pole DC motor chosen based on the power required to circulate the fluid. The motor was connected to a servo controller manufactured by ESCON-Maxon, which was linked to a PC and controlled by the ESCON Studio program to adjust the impeller’s rotational speed.

The motor, flow meters, and pressure sensors were connected to an acquisition card that oversaw communication between the electronic components and the control system (Labview software 2020 Q3). Two flow meters were used in this study, the Atrato Series Ultrasonic flow meter for flow rates from 0.002–0.500 L/min, and the RS PRO flow meter for flow rates from 0.05–10 L/min (4). Two pressure sensors, type JUMO MIDAS SI (5), were used at the inlet and outlet of the pumps. The NI USB-6008 acquisition card was utilized to connect all the sensors. Valves (6) located between the pump and flow meter were utilized to adjust the flow rate. Each test was repeated three times to verify reproducibility.

## 3. Results

### 3.1. Experimental Hydraulic Performances

**Figure 7:**
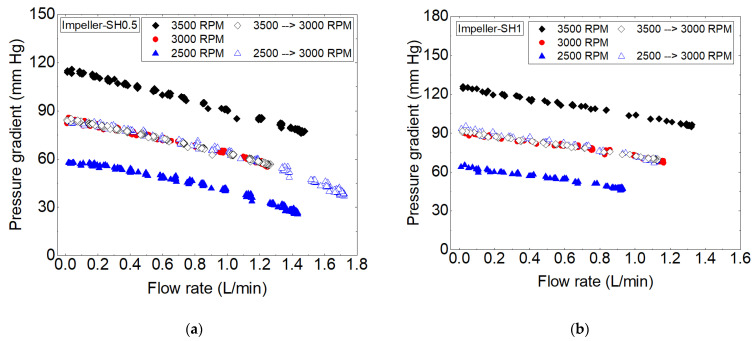
Experimental hydraulic performances of the pumps with (**a**) impeller SH0.5 and (**b**) impeller SH1 at different rotational speeds.

The hydraulic performances of the two prototypes are shown in Figure 7 for three rotational speeds: 2500, 3000, and 3500 rpm. All data points were also plotted at a common speed of 3000 rpm to facilitate comparison. Regardless of the flow rate, no significant differences in similarity were observed, demonstrating the accuracy and repeatability of the measurements taken for these flow regimes. The target flow-pressure point of 0.5 L/min and 80 mm Hg was achieved at 3000 rpm and 2900 rpm for SH0.5 and SH1, respectively.

This slight difference in angular speed for the same flow rate is due to the difference in designs of the rotor’s blades. In practice, the value of 2900 rpm was determined by scaling the speed with similarity coefficients, based on the measured pressure-flow values for the SH1 pump at a given reference rotation speed of N = 3000 rpm.

### 3.2. Overall CFD Results and Experimental Comparison

**Figure 8:**
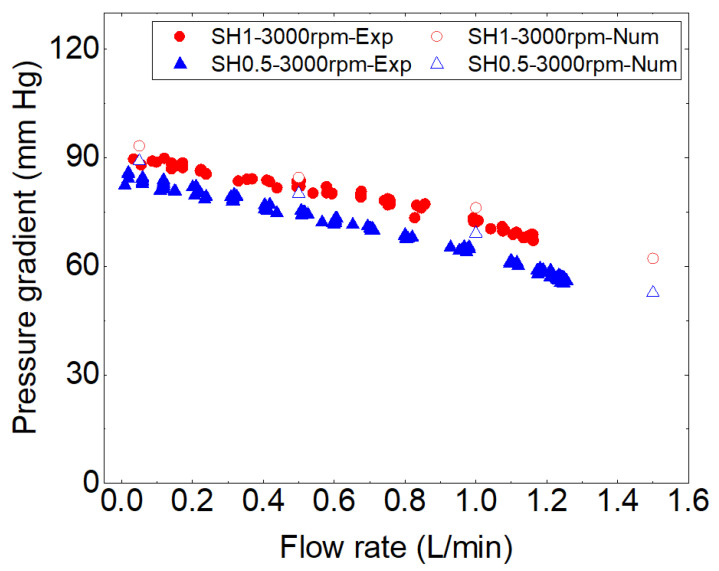
Comparison of experimental and numerical hydraulic performances for two prototypes operating at 3000 rpm.

Figure 8 shows a comparison between the experimental and numerical results, specifically the pressure gradient as a function of flow rate for the two cases examined. In both cases, a reasonable agreement is observed for all compared flow rates. Therefore, we can confidently use CFD simulations to further analyze the hemocompatibility of these two pump variants.

### 3.3. Hemocompatibility Analysis

**Figure 9:**
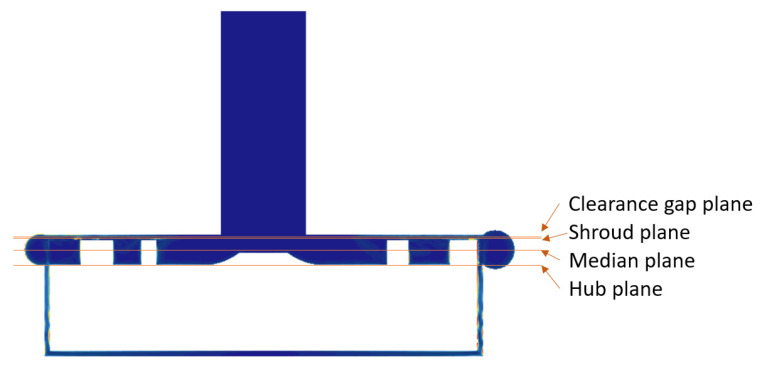
Four planes showing the details of the SSS.

**Figure 10:**
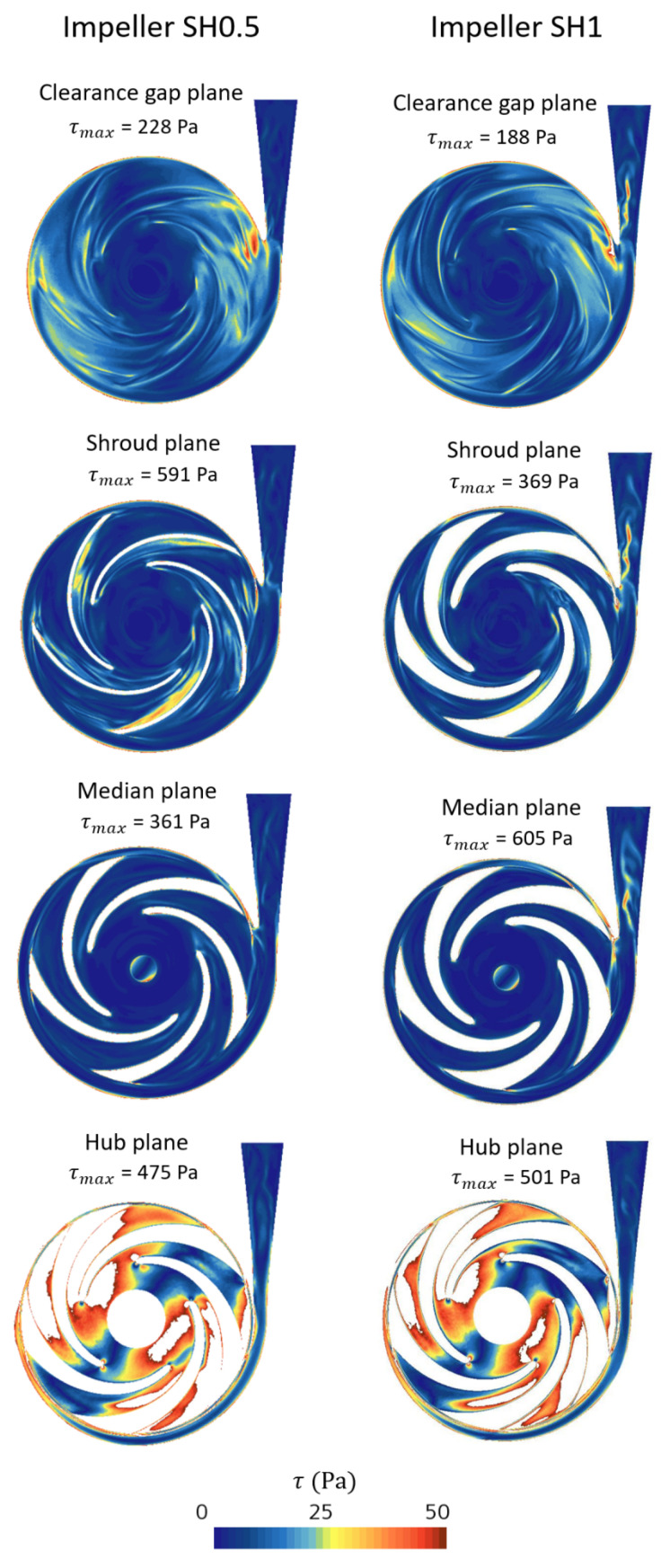
SSS fields of the two pumps at four planes.

To illustrate the magnitudes of SSS fields and compare the behavior of the two prototypes, four planes perpendicular to the axis of rotation were chosen within the fluid volume. These planes are located at the mid-clearance, blade shroud, mid-blade width, and blade-hub positions, as depicted in Figure 9. Figure 10 shows a high magnitude of SSS at the blade shroud and clearance planes. Within these two planes, the SH0.5 pump reaches higher maximum magnitude values, with an SSS magnitude approximately twice that of the SH1 pump in the blade shroud plane.

**Figure 11:**
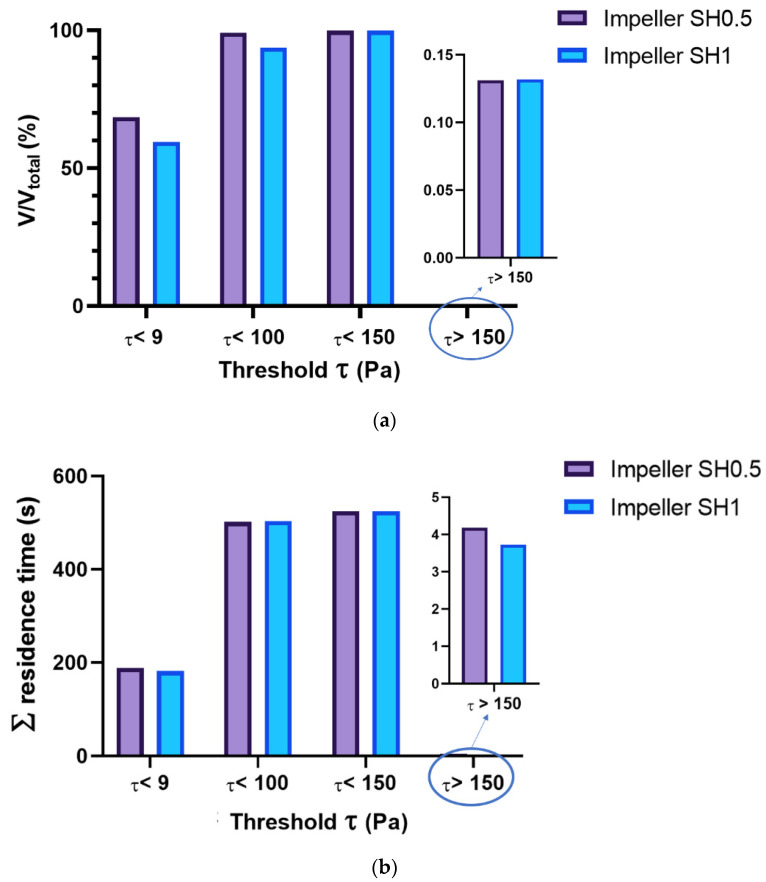
(**a**) Volume fractions and (**b**) the sum of cell residence times, subjected to the specific shear stress thresholds.

In Figure 11a, the volume of fluid exposed to various levels of SSS is shown for both prototypes to provide an overview of the SSS generated throughout the entire fluid domain. The impeller SH0.5 results in a larger volume of fluid exposed to each specific SSS threshold. Specifically, for pump SH0.5, approximately 99.1% of the fluid volume is exposed to SSS below 100 Pa, while for pump SH1, this value is around 93.8%. It is worth noting that regions of very high SSS (>150 Pa) are highly localized and constitute less than 1 mm³ for both impellers, which is less than 0.15% of the total fluid volume of the rotor. By comparison, the volumes of high scalar SSS found in the blood pumps of Fraser et al. [24] range from 0.3% to 1.4% for axial pumps and from 0.1% to 0.3% for centrifugal pumps. Thamsen et al. [37] show that the regions of high shear stress (>150 Pa) are below 0.025 mL for both HeartMate II and HVAD devices, representing approximately 1.4% and 1% of the total fluid volume, respectively. Figure 11b shows the residence time exposed to each threshold stress. The results show that there is a difference in the exposition times for the two Pumps SH0.5 and SH1. One can note that the residence time for pump SH0.5 is higher than SH1, which can increase the possible blood damage. Thamsen et al. [37] indicated that the residence time exposed to the shear stress above 150 Pa for the HeartMate II and HVAD are approximately 50 s and 28 s, respectively.

**Figure 12:**
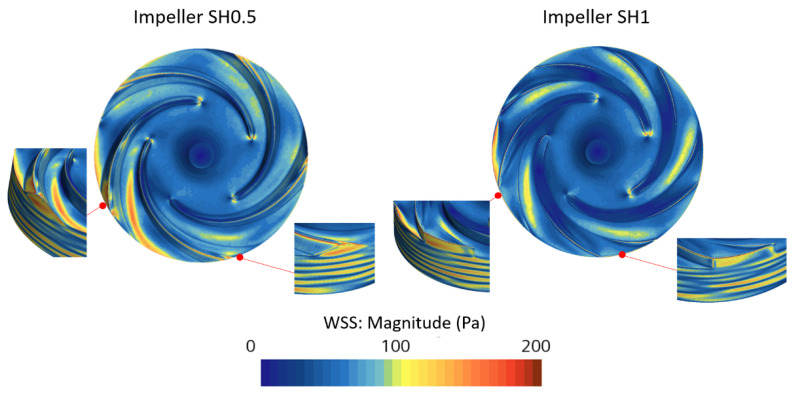
WSS magnitude for the two pumps.

The wall areas where the magnitude of WSS is greater than 100 Pa correspond to 2.79 × 10^−4^ and 1.95 × 10^−4^ m² for SH0.5 and SH1, respectively. The maximum magnitude is higher for SH0.5, with a value of 883 Pa compared to 747 Pa for SH1. As depicted in Figure 12, for both impellers, the highest magnitudes of WSS occur near the trailing edge on both the suction and pressure sides.

**Figure 13:**
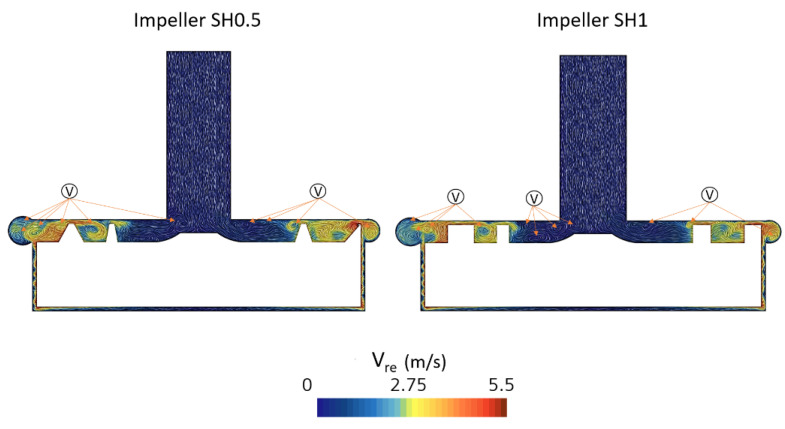
Relative velocity vector in the clearance gap for two pumps with impeller SH0.5 and SH1 (v in this figure indicates the vortices).

Figure 13 shows the relative velocity fields in the clearance gap. The SH0.5 pump has more extensive swirling zones than the SH1 pump. The thinner shroud thickness in this area for the SH0.5 impeller promotes this increased recirculation, which is more noticeable towards the trailing edge.

**Figure 14:**
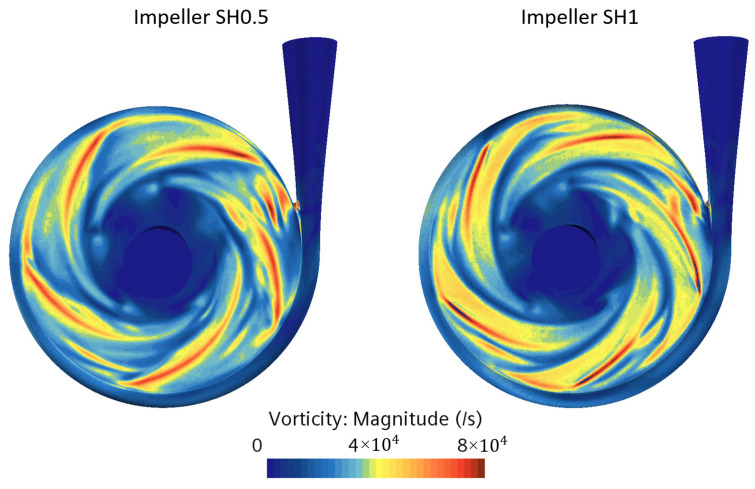
Vorticity magnitude in the fluid volume for the two pumps with impeller SH0.5 and SH1.

Regarding the vorticity calculations, the volumes occupied by vorticity magnitudes greater than the threshold of 4000/s are 4620 and 1430 mm³ for pumps SH0.5 and SH1, respectively. The maximum magnitudes are 185,000/s and 178,000/s for pumps SH0.5 and SH1, respectively. Figure 14 shows the vorticity fields in the whole fluid volume, and Table 2 summarizes the maximum magnitude observed in four selected planes. Once again, as with the SSS, it is observed that the vorticity magnitude is higher in the housing and clearance planes for pump SH0.5 compared to pump SH1.

## 4. Discussion

Heart failure can be treated with mechanical circulatory systems or assist devices, but their hemocompatibility must be studied to ensure their effectiveness [42]. The hydraulic results obtained in this study through simulation show good correlation with experimental results. The SH1 pump required 100 fewer rpm than the SH0.5 pump to achieve the same operating point. However, at this preliminary stage of the study, a question arises about the influence of the chosen assumptions regarding the input/output boundary conditions. Previous studies [28,29] have shown that pulsatile flow can reduce pressure gradient and shear stress on the left ventricular wall, leading to the development of valveless pulsatile pumps, which have been shown to be effective. Conversely, other studies [43] have demonstrated that continuous flow pumps can maintain the hemodynamic conditions of the heart’s pulsatile flow at any heart rate. Continuous-flow left ventricular assist devices can effectively unload the ventricle and increase cardiac output. However, continuous blood suction from the ventricular apex can alter hemodynamics, and obstruction of the left ventricular assist device in the ventricle can affect the distribution of vorticity, creating a stagnant region at the ventricular apex. The use of certain pumps can lead to the degradation of red blood cells due to higher shear stress [35], which has been linked to blood dysfunction and hemolysis, presenting a major limitation for these devices. Threshold shear stress values have been identified to detect dysfunction [44,45], with values of 9, 50, and 150 Pa corresponding to degradation of Willebrand factor, platelet activation, and red blood cell rupture (hemolysis) [24,37,46,47,48]. To improve hemocompatibility and reduce dysfunction, designers have focused on the geometric parameters of pumps, such as the wrap angle, number of blades, blade thickness, impeller diameter, and gap size [49,50,51,52,53,54]. However, increasing the number of blades can result in higher SSS levels. Additionally, high vorticity levels have been associated with an increased risk of thrombosis, with a vorticity level of approximately 1000 s^−1^ potentially indicating a higher risk. Nonetheless, these thresholds are not universally agreed upon and must be considered along with other factors in the design and use of pumps. Results from hemodynamic analyses showed that reducing blade thickness at the shroud level led to higher simulated magnitudes of vorticity and SSS, which disadvantaged the SH0.5 pump compared to the SH1 pump.

## 5. Conclusions

In this paper, we describe a preliminary prototype design study of a centrifugal pump, ultimately, for unloading the human heart from the left atrium, with the hope of treating heart failure with preserved ejection fraction (HFpEF). We designed and manufactured a prototype with two different impeller geometries and an identical volute. Hydraulic performance tests on an internal in vitro circulatory loop, as well as numerical simulations, were performed to evaluate and compare the performance of the two prototypes, specifically in terms of meeting flow rate–pressure gradient specifications and hemocompatibility, analyzing SSS, WSS, and vorticity fields. The results showed that both prototypes, SH0.5 and SH1, can provide the specified atrial unloading (0.5 L/min and 80 mm Hg) and that the SH1 pump appears to have an advantage over the SH0.5 pump in terms of hemocompatibility (lower simulated magnitudes of vorticity and SSS). The next step in our research project consists of two work packages: (i) improving the prototypes by including the specified magnetically levitated electric motorization, and (ii) conducting in vitro hemodynamic tests with real blood. This second step will allow us to verify the results of the pre-design and approach the following phases with a better understanding.

## Figures and Tables

**Table 1:** Main geometrical features of the impellers.

	Impeller SH0.5	Impeller SH1
Impeller CAD	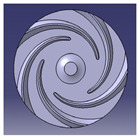	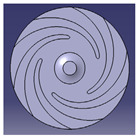
δ_LE-Hub_	1.00	1.00
δ_TE-Hub_	3.68	3.68
δ_LE-Shroud_	0.5	1.00
δ_TE-Shroud_	0.5	3.68
β_1_	15	15
Β_2_	24	24
Z	5	5
D_1_	17.5	17.5
D_2_	35	35
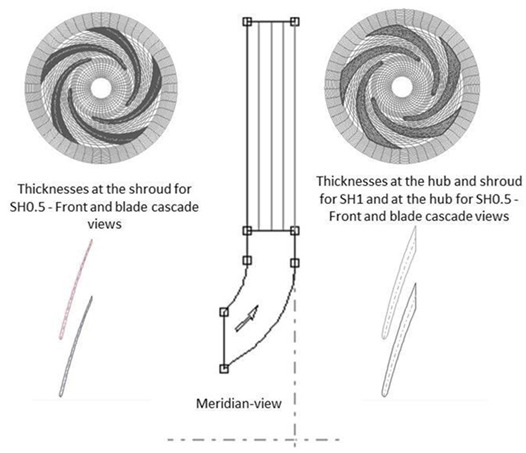		

**Table 2:** Maximum vorticity at four planes of two prototypes.

Plan Name	Impeller SH0.5	Impeller SH1
Clearance gap plane	74 × 10^3^	52 × 10^3^
Shroud plane	177 × 10^3^	161 × 10^3^
Median plane	86 × 10^3^	157 × 10^3^
Hub plane	108 × 10^3^	116 × 10^3^

## Data Availability

The data presented in this study are available on request from the corresponding author.

## Nomenclature

b_2_	impeller outlet width, mm
D_1_	impeller inlet diameter, mm
D_2_	impeller outlet diameter, mm
HFpEF	heart failure with preserved ejection fraction
N	rotation speed, rpm
Q	flow rate, L/min
V/Vt	volume fraction, %
V_rel_	relative velocity, m/s
Vorticity	vorticity magnitude, s^−1^
WSS	Wall Shear Stress, Pa
Z	blades number
β_1_	blade inlet angle, °
β_2_	blade outlet angle, °
δ_LE_	leading edge blade thickness, mm
δ_TE_	trailing edge blade thickness, mm
ΔP	pressure gradient, mm Hg
τ	Scalar Shear Stress (SSS), Pa
